# The Influence of Personal Well-Being on Learning Achievement in University Students Over Time: Mediating or Moderating Effects of Internal and External University Engagement

**DOI:** 10.3389/fpsyg.2017.02287

**Published:** 2018-01-09

**Authors:** Lu Yu, Daniel T. L. Shek, Xiaoqin Zhu

**Affiliations:** ^1^Department of Applied Social Sciences, The Hong Kong Polytechnic University, Hong Kong, China; ^2^Department of Social Work, East China Normal University, Shanghai, China; ^3^Kiang Wu Nursing College of Macau, Macau, China; ^4^Hong Kong Institute of Service Leadership and Management Limited, Hog Kong, China; ^5^Division of Adolescent Medicine, Department of Pediatrics, Kentucky Children's Hospital, University of Kentucky College of Medicine, Lexington, KY, United States

**Keywords:** university engagement, personal growth, academic achievement, GPA, Chinese student

## Abstract

The current study examined the relationship between students' personal well-being and their learning achievement during university study, and whether such relationship would be mediated or moderated by university engagement. A total of 434 university students from one public university in Hong Kong participated in the study. The participants completed an online survey consisting of personal well-being (cognitive behavioral competence and general positive youth development), university engagement, and learning achievement measures (personal growth, and accumulated GPA as academic achievement) at four time points with a 1-year interval. Results showed that personal well-being measured at the beginning of university study positively predicted students' personal growth and academic achievement after 3 years' study. While the internal dimensions of university engagement (academic challenge and learning with peers) showed longitudinal significant mediational effect, the external dimensions (experience with faculty and campus environment) did not have significant longitudinal moderating effect. Nevertheless, external dimensions of student engagement also showed direct effect on personal growth and academic achievement. The long-standing positive effects of personal well-being on university engagement and subsequently, learning achievement during university years call for more attention to the promotion of holistic development among university students in Hong Kong.

## Introduction

University students have been identified as an “at-risk” population, because the age at which most young people start higher education coincides with the age of onset of a range of problem behaviors (e.g., substance abuse and internet addiction) and mental health problems (e.g., depression and anxiety; Wynaden et al., 2013). These psychosocial problems have been progressively highlighted for not only their increased incidence and severity, but also the close link to negative quality of life, such as poor academic performance, decreased life satisfaction, and even suicidal thoughts (Eisenberg et al., 2009). In addition, the transition to higher education itself is a great challenge, which may cause physical and psychological distress and negatively affect the quality of university students' academic life (Tobolowsky, 2008; Wynaden et al., 2013).

Yet, students who can cope effectively and function adaptively in university would flourish amidst these challenges (Stamp et al., 2015). Whether students can function optimally in challenging circumstances is a result of complex interactions between individual (e.g., personal attributes and biological condition), social, environmental, and cultural factors. Most importantly, protective factors can modify students' response to challenges and buffer them from the negative influences (Bouteyre et al., 2007; Burris et al., 2009). Thus, to help students deal with developmental and transitional difficulties in university, and to promote their gains from higher education, it is very important to explore such protective factors. At a personal level, previous studies have shown that personal well-being such as interpersonal confidence, social and emotional skills, and self-esteem were associated with better adjustment and learning achievement (Eisenberg et al., 2009; Stamp et al., 2015). Hence, focusing on these attributes represents a promising approach to enhance university students' learning gains.

### Personal well-being and student learning achievement

Positive psychologists focus on the bright sides of adolescents and perceive all adolescents as “resources to be developed” (Lerner et al., 2003, p. 172). In particular, a positive youth development (PYD) framework has been proposed which emphasizes the importance of developing multiple psychosocial competences (e.g., cognitive competence, social emotional competence, and spirituality) in constructing a good life among youth (Catalano et al., 2012). Under this paradigm, personal well-being in terms of psychosocial competencies, such as emotional competence, resilience, and moral competence have been increasingly emphasized in recent years (Roth and Brooks-Gunn, 2003), particularly for its relationship with student academic achievement. For example, self-efficacy, social competence, and emotional skills are positively related to school performance and negatively associated with problem behaviors among adolescents and university students (Caprara et al., 2011; Polan et al., 2013). Similarly, recent meta-analytic studies concluded that intervention and prevention programs (e.g., service learning programs and social emotional learning programs) fostering students' competencies have shown positive effects on students' academic success, with small to large effect sizes (Sklad et al., 2012; Gutman and Schoon, 2015).

Despite these consistent findings, four limitations exist in the extant literature regarding the relationship between personal well-being and students' learning achievement. First, most of the findings were obtained from cross-sectional studies, which could not provide a full picture about how personal well-being may contribute to their learning effectiveness over time. Second, most research was conducted among secondary school students, while few studies focused on university students. This gap is particularly important when we realize that there are growing developmental issues and mental health problems in university students.

Third, such studies are especially scarce in the non-Western populations such as Chinese youth in Hong Kong. While adolescents are commonly taught about social, emotional, and other life skills before entering university in the Western contexts (Catalano et al., 2002; Durlak et al., 2011), systematic training on individual psychosocial competences is not well developed in Hong Kong (Shek et al., 2007a; Shek and Yu, 2011). In Chinese societies, it is commonly believed that young people will have a bright future as long as they can be admitted to a distinguished university. This traditional belief drives parents, teachers, schools, and even students themselves to place morbid emphasis on attaining high academic achievement, which is a prerequisite for students being admitted to a distinguished school at each learning stage and getting into dream university eventually.

Besides, the development of psychosocial competences among young people has been largely overlooked. In fact, with more and more psychological and behavioral problems being identified among Hong Kong youngsters (Wong et al., 2006; Song et al., 2008; Shek and Wong, 2011), we have to admit that students' prior academic development cannot guarantee their achievement in university. Although there are researchers advocating that personal well-being and psychosocial competences are critical for young people to make the most out of their university education (Shek and Wu, 2016), both public and scientific attentions to this issue have been inadequate. Therefore, to further raise the awareness of and win support for the importance of promoting holistic development among Hong Kong youth, it is essential to provide more evidence for the impact of adolescents' psychosocial competences on their subsequent learning achievement in university. Moreover, concerning the great proportion (i.e., nearly one-fifth) of Chinese people in the world's population, studying the relationship between personal well-being and student learning achievement among Chinese people is indispensable to provide answers to the universality of such relationship.

Fourth, previous literature has not fully addressed the mechanisms underlying the relationship between personal well-being and learning outcomes. It is possible that the relationship could be mediated or moderated by factors at both individual, and other ecological levels. While family factors such as parental involvement and family support have been identified as important facilitators for students' learning achievement (e.g., MacGeorge et al., 2005; Cheng et al., 2012), the role of other factors in one's undergraduate life, such as university engagement, has not been thoroughly investigated. Specifically, concerning the lack of research on university engagement in Chinese context, this question is especially relevant in the present study.

To address the above research gaps, the present study had two objectives: (1) to examine the relationship between personal well-being and student learning achievement in one Chinese community (i.e., Hong Kong) using a longitudinal research design, and (2) to explore whether and how university engagement could possibly underlie such relationship. Sections below will focus on the elaboration on the second research objective.

### Effects of university engagement

In literature, the concept of university engagement considers dimensions at individual (i.e., students themselves) and the contextual or institutional level. For example, in a widely-cited definition given by Kuh (2009a), university engagement was regarded as “the time and effort students devote to activities that are empirically linked to desired outcomes of college and what institutions do to induce students to participate in these activities” (p. 683). Similarly, researchers framed university engagement in terms of external factors (e.g., institutional factors) and internal factors (i.e., students' personal factors; Zhang et al., 2015).

Accordingly, in the large-scale national survey conducted annually in USA (i.e., National Survey of Student Engagement, NSSE) and Australia (i.e., Australasian Survey of Student Engagement, AUSSE), university students' engagement was empirically indexed by engagement indicators covering both student learning and student perception on campus resources (Kuh, 2017). The structure of engagement indicators has been reviewed recently, and now there are 10 engagement indicators, which can be further categorized into four higher-order engagement themes (NSSE, 2015). The four themes include academic challenge (e.g., higher-order learning and learning strategies), learning with peers (e.g., collaborative learning), experiences with faculty (e.g., student-faculty interaction and effective teaching practice), and campus environment (e.g., supportive environment). Conceptually speaking, while the former two higher-order themes primarily highlight student personal devotion to learning, which reflects the internal dimensions of university engagement, the latter two mainly focus on the perceived support and resources provided by peers, teachers, faculty and the institution, which could be regarded as the external or contextual dimensions of university engagement.

Ample studies have demonstrated a strong positive association between university engagement and student learning achievement, such as critical thinking, cognitive development, and academic achievement, both in Western countries (Trowler and Trowler, 2010; Fuller et al., 2011) and China (Lu et al., 2014). While it is well documented that both internal factors (e.g., learning involvement) and external factors (e.g., campus resources) could serve as facilitators of university learning achievement, their different roles in students' university study have not been systematically examined, especially for their mediating or moderating effects on the relationship between personal well-being and student learning achievement.

### Mediating effect of internal engagement

The critical role of internal engagement is well defined by college impact models (e.g., Inputs-Environment-Outputs Model; Astin, 2012), which have been commonly used to interpret causal relationships of university characteristics as well as students' diverse learning experiences to student gains and development (Pascarella et al., 2014; Kilgo et al., 2015). In general, college impact models acknowledge the influence of two types of factors on student gains. The first one was students' background and personal situation they possessed before attending college (i.e., “input”), and the second one was students' learning experiences such as their own involvement during college learning (i.e., “process”).

According to college impact models, student learning achievement is the “output” of college impact, and the so called “input” factors can either directly affect “output” or indirectly influence it via the “process” factor (e.g., Astin, 2012). Theoretically, students' personal well-being in terms of competences is among the “input” factors because well-being constitutes one aspect of students' personal situation at the beginning of university life. Likewise, student internal university engagement, which represents how they involve in and the extent they devote themselves to university learning, can in part index the “process” factor. In this sense, we could expect that students' personal well-being as the “input” will affect their learning achievement (i.e., output) through the influence of internal engagement (i.e., process). In other words, students with a high level of personal well-being may be more engaged in university learning and thus obtain greater learning gains.

Evidence has supported the positive association between personal well-being and students' internal learning engagement. For example, enhancing students' self-belief was considered as one strategy to improve student engagement in higher education (Zepke and Leach, 2010). Related findings also showed that learners who had higher self-efficacy showed a higher level of engagement (Llorens et al., 2007). Furthermore, students' self-perceived competence within learning context could facilitate their ongoing active learning (Fazey and Fazey, 2001). Therefore, students who were confident in their own competence could remain motivated and be engaged even in difficult situations.

With specific reference to the Chinese context, although the number of studies on university students' engagement and its individual differences has increased in recent years (Lu et al., 2014; Yin and Wang, 2016), these studies primarily focused on sociodemographic variables such as gender and grade level rather than personal attributes. Among the few exceptions, Siu et al. (2014) reported that psychological capital defined by resiliency, optimism, hope, and self-efficacy positively predicted undergraduates' study motivation and learning involvement in Hong Kong. In their qualitative study, Zhang et al. (2015) found that individual factors such as adaptation, communication skills, personality (e.g., confident) would impact students' learning engagement in university.

The above indirect evidence suggests a positive relationship between personal well-being and internal university engagement in Chinese context as well. This finding, in conjunction with the robust predicting effect of university engagement on learning achievement (Trowler and Trowler, 2010; Lu et al., 2014), suggests that internal dimensions of university engagement may mediate the relationship between personal well-being and learning achievement, especially when students are in a difficult transition period to higher education. This is certainly the case in Hong Kong, where the higher education reform adds extra challenges to students' transitional difficulties (Jaffee, 2012). However, little research attention has been paid to this topic. To fill this gap, the present study attempted to investigate the impact of personal well-being on student learning achievement and the potential mediating effect of internal university engagement in Hong Kong students.

### Moderating effect of external engagement

In dealing with transitional distress, while those students possessing sufficient competences as internal resources can convert stressors into opportunities for positive development, those students lacking personal well-being may suffer from adverse consequences (Caprara et al., 2011; Polan et al., 2013). However, positive school environments as external factors related to university engagement could buffer negative influence on learning achievement of various risk factors, such as family disadvantages, transition distress, and students' disruptive behaviors (Hopson and Lee, 2011; O'Malley et al., 2015). In this sense, positive experiences with faculty and a favorable campus environment may in part complement for the insufficient personal well-being, thus attenuating the relationship between lack of personal competencies and undesirable learning achievement. In other words, external dimensions of university engagement may moderate the relationship between personal well-being and university learning achievement.

Under the framework of university engagement, the positive school environment mainly relates to resources and support provided by the faculty and institution, effective teaching practices, and constructive relationships between students and teachers and faculty members. These contextual factors, as reflected in the two external university engagement themes, constitute important parts of university students' support system, which could help students better cope with difficulties and challenges encountered in university study. To illustrate, perceived social support from professors and peers were beneficial to students' self-esteem and grades (Clifton et al., 2004). Besides, social support including peer support acted as a buffer against academic stress in university (Wilks and Spivey, 2010). Therefore, the supportive university environment may create conditions for achieving favorable learning achievement. Such a favorable context could possibly add extra increments to internal assets while simultaneously counteract the negative impact of the lack of those internal resources.

The moderating effect of external dimensions of university engagement is more likely to occur in Chinese context, given that peers, teachers and the school as external factors may play even greater roles in shaping Chinese students' learning than they do in Western societies. For instance, compared with Western counterparts, Chinese students perceive a higher level of peer support in learning, and establish friendship with each other more often by doing homework or preparing for examinations together (Jia et al., 2009), which is regarded as helpful for obtaining good marks (Zhang et al., 2015). Besides, Chinese teachers form a closer “friendship” with students and are more likely to play the formal and authority role in helping students during their first 1–2 years' of university learning (Zhu et al., 2010). A more recent study also highlighted the pivotal role played by teachers in Chinese students' learning involvement and their learning achievement in university (Chi et al., 2017). Nevertheless, the hypothesized moderating effect of external dimensions of university engagement has not been empirically examined in Chinese context.

### Influence of social-demographic factors

To examine the relationship among personal well-being, university engagement, and student learning achievement, it is important to control confounding variables, such as gender and family background. Regarding gender, female students are usually found to be more engaged in learning and achieve better academic performance than male students across cultures (Lam et al., 2012). A recent study in Chinese context found that female students engaged in university learning in a more adaptive way than did their male peers (Yin and Wang, 2016). Besides, prior research found that student development varies by family characteristics. For instance, a harsh family environment (e.g., poor, non-intact family structure) were negatively associated with students' academic performance (Hopson and Lee, 2011; O'Malley et al., 2015). Furthermore, recent large-scale longitudinal studies uncovered the concurrent and longitudinal relationships between unfavorable family conditions (e.g., non-intact family structure and economic disadvantage) and poor youth development among Chinese youth (Yu and Shek, 2014; Shek and Leung, 2016). Therefore, the present study considered family intactness and family economic status as two control variables in addition to gender and age.

### The present study

To fill the current research gaps, the present study aimed to address three research questions:

1. Does students' personal well-being longitudinally predict their learning achievement?
2. Do internal dimensions of university engagement mediate the above predicting effect?
3. Do external dimensions of university engagement moderate the above predicting effect?

Based on the extant literature, we had four hypotheses presented below:

- *Hypothesis 1*: University students' personal well-being would predict their learning achievement positively.
- *Hypothesis 2*: The above-hypothesized predictive effect would be partially mediated by the internal dimensions of university engagement indexed by academic challenge and learning with peers.
- *Hypothesis 3*: The external dimensions of university engagement would be positively related to students' learning outcomes.
- *Hypothesis 4*: The external dimensions of university engagement would also moderate the relationship between students' personal well-being and their learning outcomes.

**Figure 1:**
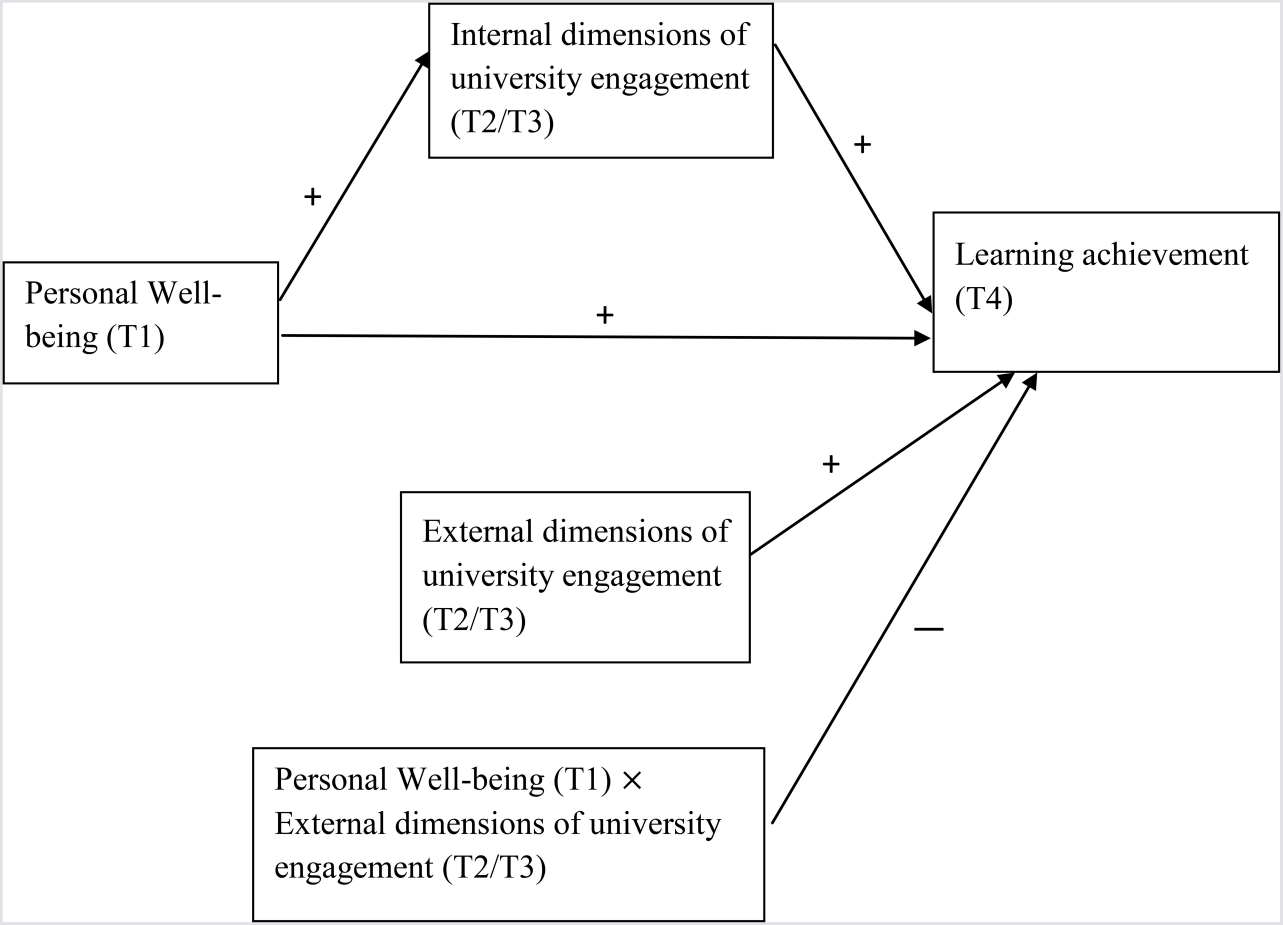
Hypothesized relationships among personal well-being, learning achievement, internal, and external dimensions of university engagement. T1 = Time 1; T2 = Time 2; T3 = Time 3; T4 = Time 4.

Figure 1 outlines the relationships between personal well-being, learning outcomes, and university engagement.

## Methods

### Participants and procedures

The present study was part of a longitudinal project commencing in the academic year of 2012–2013, the first year when public universities in Hong Kong started implementing the 4-year undergraduate curricula. The project was approved by the Human Subjects Ethics Sub-committee (HSESC) (or its Delegate) of the authors' university. In this project, 650 first-year students in one public university in Hong Kong were randomly selected and invited via email or phone calls to join a longitudinal online survey aiming to investigate students' changes under the new undergraduate curriculum. Consent from students was obtained and they were well informed that they could withdraw from the study whenever they want, and that any information they provided would be kept strictly confidential and only used for research purposes.

Time 1 data collection was carried out in November 2012, 2 months after students' enrollment in the university. Out of 650 invited students, 543 completed the survey suggesting a valid response rate of 83.54%. These students were followed up also in November in the following 3 consecutive years and invited to complete the same online survey. Across four time points (i.e., Time 1, Time 2, Time 3, and Time 4), 434 participants (*M*_*age*_ = 18.13, *SD*_*age*_ = 0.54 at Time 1) were successfully matched, suggesting an acceptable total attrition rate of 20.07% across 3 years. All participants were Asian, while 316 (72.8%) were Hong Kong local students, 105 (24.3%) were mainland Chinese students, 13 (3.0%) were students from other Asian countries. There were 266 female students (61.29%) and 168 male students (38.71%).

Comparison between the matched sample (*n* = 434) and those dropouts of the survey after the first wave of data collection (*n* = 109) did not yield any significant differences regarding demographic information (i.e., gender composition, age, ethnicity, and place of birth), family background, and personal well-being measured at Time 1. Therefore, the matched sample (*n* = 434) was used as the basis of the present analyses.

### Instruments

The questionnaires used in the project included multiple measures. The current study focused on the relationship among personal well-being, engagement, and learning outcomes and the related measures are described below. To facilitate students' completion of questionnaires, all items were phrased in both English and Chinese.

#### Learning achievement measures

Personal growth was measured at Time 2, Time 3, and Time 4 in the longitudinal project. In the present study, personal growth measured at Time 4 was used as one indicator of student learning achievement after 3 years of study. Participants reported their own growth since attending the university using a 10-item scale adopted from US NSSE. These items covered such aspects as written and oral communication skills, social skills, general knowledge, critical thinking, intellectual skills, problem solving, ethical development, and civic responsibility. This measure represented a value-added approach for learning outcome assessment incorporated in NSSE (Kuh, 2009b), and can be seen as an important complement to direct measures of learning, such as grade point average (GPA). Besides, the 10 items were well designed to meet certain conditions under which students' own estimates for growth are valid and reliable (Kuh, 2009b). A 4-point Likert scale (1 = strongly disagree; 4 = strongly agree) was adopted with higher scores suggesting greater growth. Student personal growth was indexed by the average score across the 10 items. Across all participants, mean score of personal growth was 2.72 (*SD* = 0.52). The 10-item scale showed good reliability with Cronbach's α of 0.90.

Academic achievement was another indicator of learning achievement. Students reported their GPA in past year since Time 2 data collection. Students' GPA in each year was recoded using the following scheme: D or lower = 1; D+ = 2; C− = 3; C = 4; C+ = 5; B− = 6; B = 7; B+ = 8; A− = 9; A = 10; A+ = 11, with a larger number representing a better score. Then an average score of GPA (i.e., accumulated GPA) across 3 years was computed for each participant. The accumulated GPA used to indicate students' academic achievement at Time 4 ranged between 3.67 and 10.33, with a mean score of 7.36 (*SD* = 1.07).

#### University engagement

Engagement indicators developed in NSSE were used to measure students' engagement during the past year. In NSSE (2015), there were 47 items mapped into 10 engagement indicators to assess student involvement in high levels of learning and their perception on campus resources. Besides, these 10 engagement indicators can be further classified into 4 higher-order themes: (1) “academic challenge” which included four engagement indicators (i.e., reflective and intergrative learning, higher order learning, quantitative reasoning, and learning strategies); (2) “learning with peers” that consisted of two engagement indicators (i.e., collaborative learning and discussion with diverse others); (3) “experience with faculty” which included two engagement indicators (i.e., student faculty interaction and effective teaching practice; and (4) “campus environment” which also included two engagement indicators (i.e., quality of interaction and supportive environment). As already mentioned before, “academic challenge” and “learning with peers” were internal dimensions while “experience with faculty” and “campus environment” were external dimensions of university engagement. A 4-point scale was adopted for all items, and an average score across all items in each engagement indicator was first calculated. The higher-order theme was computed as the mean score of standardized values of the corresponding engagement indicators. In the present study, the four higher-order themes obtained at Time 2 and Time 3 were used. All engagement indicator measures demonstrated good internal reliability with Cronbach's α ranging between 0.70 and 0.90.

#### Personal well-being

Students' personal well-being was defined in terms of positive youth development competencies (Shek et al., 2017), and measured using the Chinese Positive Youth Development Scale (CPYDS) developed by Shek et al. (2007b). The original CPYDS contained 15 subscales corresponding to 15 positive development constructs (e.g., resilience, social competence, and self-determination) summarized by Catalano et al. (2002). The 90-item CPYDS has shown sound validity and reliability in measuring Chinese adolescents' personal positive attributes in previous studies (e.g., Sun and Shek, 2013). In the longitudinal project, concerning the length of the whole questionnaire, 8 subscales measuring attributes directly related to desired university graduate attributes identified in the original project were adopted (Shek et al., 2015). The eight subscales include cognitive competence, emotional competence, behavioral competence, social competence, self-determination, self-efficacy, resilience, and moral competence, which can be used to form two composite scores: cognitive-behavioral competence and general positive youth development attributes (general PYD). The cognitive-behavioral competence score was calculated as the average score of three subscales of cognitive competence, behavioral competence, and self-determination while the general PYD score was calculated based on the mean score of other four subscales of resilience, social competence, emotional competence, and moral competence (Shek and Ma, 2010). In the present study, cognitive-behavioral competence and general PYD at Wave 1 were employed as indicators of students' baseline personal well-being. A 6-point Likert scale was applied for all items with a higher score indicating a higher level of personal well-being. All scales used in the present study showed acceptable to good reliability with Cronbach's α ranging from 0.64 to 0.85.

#### Control variables

Besides demographic information such as gender and age, control variables also included family intactness and family economic status. Family intactness was defined as marital status of participants' parents. A total of 370 (85.25%) participants who reported that their parents were in the first marriage were categorized into intact family group, and 64 (14.75%) participants whose parents were divorced, separated or in their second marriage were in non-intact family group. Participants' family economic status was determined by whether his/her family received Hong Kong Government welfare (i.e., Comprehensive Social Security Assistance Scheme, CSSA) before entering university. A total of 38 (8.80%) participants whose family received CSSA prior to university were categorized as having economic disadvantage and other 386 (88.9%) participants whose family did not receive CSSA were regarded as not having economic disadvantage.

### Data analysis plan

We first conducted a correlational analysis among all related variables. To test the first and second hypotheses, the multiple mediator model (Preacher and Hayes, 2008) was utilized to examine the predictive effect of cognitive-behavioral competence and general PYD at Time 1 (i.e., independent variables) on learning outcomes at Time 4 (i.e., dependent variables), as well as the mediating effects of internal university engagement (i.e., “academic challenge” and “learning with peers”) at Time 2 or Time 3. In the current study, 5,000 bootstrap samples were used. Then, several multiple regression analyses were performed to test the third and fourth hypotheses by entering external dimensions of engagement as well as their interactions with personal well-being as predictive variables.

## Results

Correlation coefficients among key variables are depicted in Table 1. Two indicators of students' personal well-being (i.e., cognitive-behavioral competence and general PYD) at Time 1 were both significantly and positively associated with indicators of university engagement (i.e., academic challenge, learning with peers, experience with faculty, and campus environment) at Time 2 and Time 3, as well as two learning achievement indicators (i.e., personal growth and academic achievement) at Time 4. Besides, university engagement measures were also positively related to students' learning achievement indicators. The findings basically supported Hypothesis 1 and Hypothesis 2.

**Table 1:** Correlations among variables.

**Variables**		**1**	**2**	**3**	**4**	**5**	**6**	**7**	**8**	**9**	**10**	**11**	**12**	**13**	**14**	**15**
1.	T1 Age	–														
2.	Gender^a^	−0.001	–													
3.	T1 FI	−0.01	−0.01	–												
4.	T1 FES	0.14^**^	0.01	0.28^***^	–											
5.	T1 CBC	−0.06	0.09	0.08	−0.05	–										
6.	T1 GPYD	−0.08	−0.08	0.04	−0.06	0.73^***^	–									
7.	T2 AC	−0.10^*^	0.07	0.10^*^	−0.05	0.26^***^	0.25^***^	–								
8.	T2 LWP	−0.03	0.06	0.05	−0.03	0.17^***^	0.22^***^	0.57^***^	–							
9.	T2 EWF	−0.02	0.07	0.09	−0.07	0.17^***^	0.18^***^	0.63^***^	0.42^***^	–						
10.	T2 CE	−0.06	−0.07	0.13^**^	−0.13^**^	0.30^***^	0.34^***^	0.39^***^	0.28^***^	0.41^***^	–					
11.	T3 AC	−0.02	−0.09	0.02	−0.07	0.27^***^	0.27^***^	0.43^***^	0.30^***^	0.28^***^	0.31^***^	–				
12.	T3 LWP	0.03	0.03	0.05	−0.06	0.22^***^	0.23^***^	0.26^***^	0.45^***^	0.26^***^	0.27^***^	0.56^***^	–			
13.	T3 EWF	0.09	−0.10^*^	0.03	−0.03	0.17^***^	0.20^***^	0.26^***^	0.23^***^	0.35^***^	0.38^***^	0.32^***^	0.47^***^	–		
14.	T3 CE	0.08	−0.11^*^	0.08	−0.10^*^	0.32^***^	0.37^***^	0.24^***^	0.18^***^	0.30^***^	0.56^***^	0.50^***^	0.47^***^	0.60^***^	–	
15.	T4 PG	0.01	−0.05	0.05	0.01	0.25^***^	0.28^***^	0.30^***^	0.28^***^	0.32^***^	0.32^***^	0.33^***^	0.26^***^	0.35^***^	0.38^***^	–
16.	T4AA	−0.08	−0.002	0.06	−0.07	0.26^***^	0.26^***^	0.30^***^	0.15^**^	0.22^***^	0.30^***^	32^***^	0.21^***^	0.22^***^	0.32^***^	0.23^***^

a *: −1 = Female; 1 = male. T1 = Time 1; T2 = Time 2; T3 = Time 3; T4 = Time 4. FI, Family intactness (−1 = not intact, 1 = intact); FES, Family economic status (−1 = having economic disadvantage, 1 = without economic disadvantage); CBC, Cognitive-behavioral competence; GPYD, General positive youth development attribute; AC, Academic challenge; LWP, Learning with peers; EWF, Experience with faculty; CE, Campus environment; AA, Academic achievement; PG, Personal growth*;

* *p < 0.05*;

** *p < 0.01*;

*** *p < 0.001*.

Control variables were not significantly correlated with learning achievement. Besides, there was no clear pattern of correlations between control variables and university engagement. Specifically, age showed a negative correlation with academic challenge at Time 2, but not at other time points. Female students perceived experience with faculty and campus environment more positively than did male students, only at Time 3; and no gender difference was found at other waves. In addition, family intactness was positively associated with academic challenge and perceived campus environment only at Time 2, while family economic status was negatively related to perceived campus environment (i.e., students without family economic disadvantage perceived less supportive campus environment) at Time 2 and Time 3.

Results of mediation effect analyses are depicted in Tables 2, 3. Results showed significant longitudinal predicting effects of cognitive-behavioral competence and general PYD on learning achievement measures (i.e., personal growth and academic achievement) as reflected by the significant total effect of independent variables on dependent variables.

**Table 2:** Mediation effect analyses with internal engagement at Time 2 as the mediator (*N* = 425–427).

**Regression models summary**	**DV: Personal growth (Time 4)**						**DV: Academic achievement (Time 4)**					
	**IV: Time 1 Cognitive-behavioral competence**			**IV: Time 1 General PYD**			**IV: Time 1 Cognitive-behavioral competence**			**IV: Time 1 General PYD**		
	***B***	***SE***	***t***	***B***	***SE***	***t***	***B***	***SE***	***t***	***B***	***SE***	***t***
Total effect of IV on DV	0.16	0.03	5.42^***^	0.21	0.03	6.11^***^	0.33	0.06	5.43^***^	0.38	0.07	5.43^***^
**IV TO MEDIATORS**												
Time 2 AC	0.22	0.04	5.16^***^	0.25	0.05	5.21^***^	0.22	0.04	5.26^***^	0.26	0.05	5.25^***^
Time 2 LWP	0.16	0.05	3.40^***^	0.24	0.05	4.52^***^	0.17	0.05	3.49^***^	0.24	0.05	4.53^***^
**DIRECT EFFECTS OF MEDIATORS ON DV**												
Time 2 AC	0.12	0.04	3.03^**^	0.12	0.04	3.13^**^	0.38	0.08	4.66^***^	0.40	0.08	4.83^***^
Time 2 LWP	0.10	0.04	2.82^**^	0.09	0.04	2.51^*^	−0.06	0.07	−0.74	−0.07	0.07	−1.00
Direct Effect of IV on DV	0.12	0.03	4.04^***^	0.15	0.03	4.57^***^	0.26	0.06	4.16^***^	0.30	0.07	4.22^***^
**Meditation effect summary**	**Point estimate**	**Bootstrapping (BC 95% CI)**		**Point estimate**	**Bootstrapping (BC 95% CI)**		**Point estimate**	**Bootstrapping (BC 95% CI)**		**Point estimate**	**Bootstrapping (BC 95% CI)**	
		**Lower**	**Upper**		**Lower**	**Upper**		**Lower**	**Upper**		**Lower**	**Upper**
Total	0.04	0.02	0.07	0.05	0.03	0.09	0.08	0.03	0.14	0.08	0.03	0.15
Time 2 AC	0.03	0.01	0.05	0.03	0.01	0.06	0.09	0.04	0.15	0.10	0.05	0.17
Time 2 LWP	0.02	0.01	0.04	0.02	0.01	0.05	−0.01	−0.04	0.01	−0.02	−0.06	0.02

*Age, gender, family intactness, and family economic status were controlled. IV, Independent variable; DV, Dependent variable; PYD, General positive youth development; AC, Academic challenge; LWP, Learning with peers; BC, Bias corrected; CI, Confidence interval*;

* *p < 0.05*;

** *p < 0.01*;

*** *p < 0.001*.

**Table 3:** Mediation effect analyses with internal engagement at Time 3 as the mediator (*N* = 425–427).

**Regression models summary**	**DV: Personal growth (Time 4)**						**DV: Academic achievement (Time 4)**					
	**IV: Time 1 Cognitive-behavioral competence**			**IV: Time 1 General PYD**			**IV: Time 1 Cognitive-behavioral competence**			**IV: Time 1 General PYD**		
	***B***	***SE***	***t***	***B***	***SE***	***t***	***B***	***SE***	***t***	***B***	***SE***	***t***
Total effect of IV on DV	0.16	0.03	5.42^***^	0.21	0.03	6.11^***^	0.33	0.06	5.43^***^	0.38	0.07	5.43^***^
**IV TO MEDIATORS**												
Time 3 AC	0.26	0.04	6.08^***^	0.28	0.05	5.67^***^	0.26	0.04	6.03^***^	0.27	0.05	5.61^***^
Time 3 LWP	0.21	0.04	4.75^***^	0.25	0.08	4.92^***^	0.21	0.04	4.77^***^	0.25	0.05	4.89^***^
**DIRECT EFFECTS OF MEDIATORS ON DV**												
Time 3 AC	0.16	0.04	4.12^***^	0.16	0.04	4.15^***^	0.36	0.08	4.48^***^	0.37	0.08	4.59^***^
Time 3 LWP	0.06	0.04	1.67	0.06	0.04	1.52	0.04	0.08	0.48	0.03	0.08	0.40
Direct Effect of IV on DV	0.11	0.03	3.58^***^	0.15	0.03	4.38^***^	0.23	0.06	3.75^***^	0.27	0.07	3.86^***^
**Meditation effect summary**	**Point estimate**	**Bootstrapping (BC 95% CI)**		**Point estimate**	**Bootstrapping (BC 95% CI)**		**Point estimate**	**Bootstrapping (BC 95% CI)**		**Point estimate**	**Bootstrapping (BC 95% CI)**	
		**Lower**	**Upper**		**Lower**	**Upper**		**Lower**	**Upper**		**Lower**	**Upper**
Total	0.05	0.03	0.09	0.06	0.03	0.09	0.10	0.05	0.16	0.11	0.06	0.18
Time 3 AC	0.04	0.02	0.08	0.04	0.02	0.08	0.09	0.05	0.16	0.10	0.05	0.18
Time 3 LWP	0.01	−0.002	0.03	0.01	−0.004	0.04	0.01	−0.03	0.04	0.01	−0.03	0.05

*Age, gender, family intactness, and family economic status were controlled. IV, Independent variable; DV, Dependent variable; PYD, positive youth development; AC, Academic challenge; LWP, Learning with peers; BC, Bias corrected; CI, Confidence interval*;

*** *p < 0.001*.

**Figure 2:**
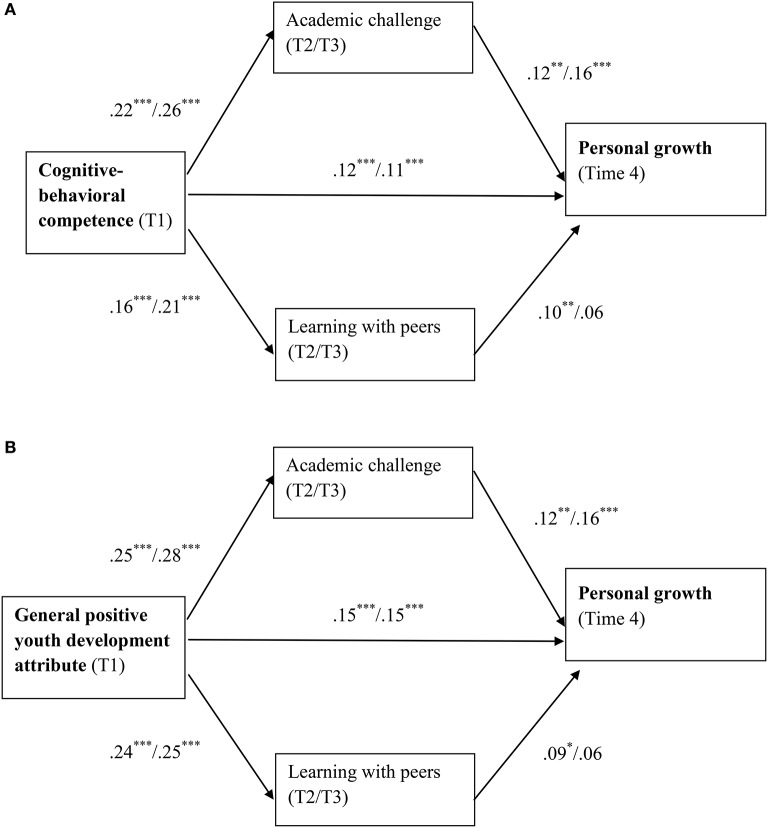
**(A)** Relationships among cognitive-behavioral competence, personal growth, and internal dimensions of university engagement. T1 = Time 1; T2 = Time 2; T3 = Time 3; T4 = Time 4. The regression coefficients shown in the figure are unstandardized values with values before and after slash representing using Time 2 and Time 3 internal dimensions of university engagement, respectively. **(B)** Relationships among general positive youth development attribute, personal growth, and internal dimensions of university engagement. T1 = Time 1; T2 = Time 2; T3 = Time 3; T4 = Time 4. The regression coefficients shown in the figure are unstandardized values with values before and after slash representing using Time 2 and Time 3 internal dimensions of university engagement, respectively. ^*^p < 0.05; ^**^p < 0.01; ^***^p < 0.001.

For students' personal growth at Time 4, the longitudinal predicting effect of Time 1 cognitive-behavioral competence was partially mediated by Time 2 internal engagement dimensions of academic challenge (*B* = 0.03, 95% CI = [0.01, 0.05]) and learning with peers (*B* = 0.02, 95% CI = [0.01, 0.04]; See Table 2). Time 3 academic challenge (*B* = 0.04, 95% CI = [0.02, 0.08]) showed similar partial mediating effect, while Time 3 learning with peers (*B* = 0.01, 95% CI = [−0.002, 0.03]) did not (See Table 3). For the predicting effect of general PYD on personal growth, the internal dimensions of university engagement demonstrated a similar pattern of mediating effects. Figures 2A,B depict the mediating effects of internal dimensions of university engagement regarding students' personal growth.

**Figure 3:**
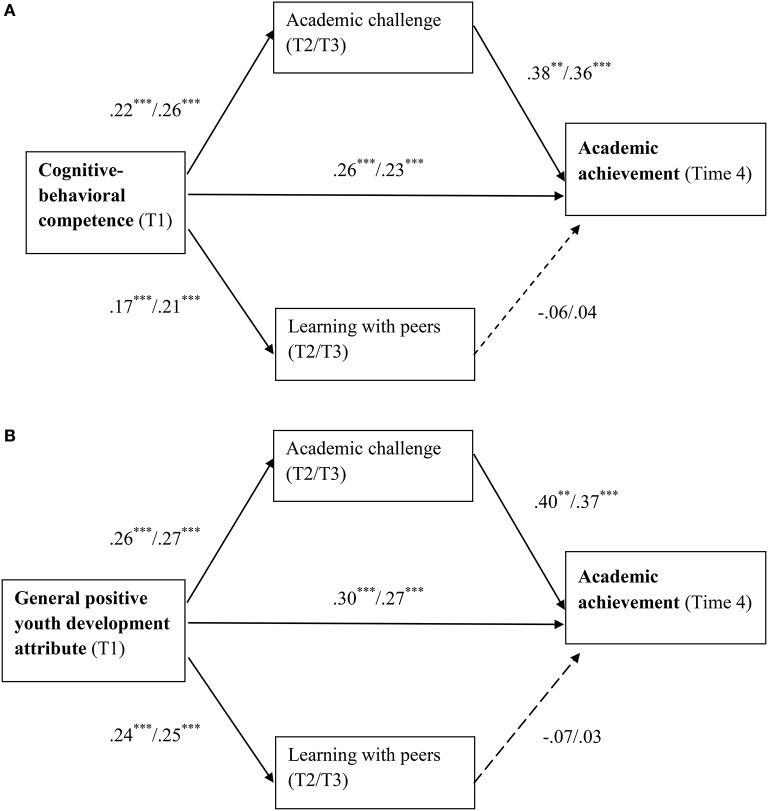
**(A)** Relationships among cognitive-behavioral competence, academic achievement, and internal dimensions of university engagement. T1 = Time 1; T2 = Time 2; T3 = Time 3; T4 = Time 4. The regression coefficients shown in the figure are unstandardized values with values before and after slash representing using Time 2 and Time 3 internal dimensions of university engagement, respectively. **(B)** Relationships among general positive youth development attribute, academic achievement, and internal dimensions of university engagement. T1 = Time 1; T2 = Time 2; T3 = Time 3; T4 = Time 4. The regression coefficients shown in the figure are unstandardized values with values before and after slash representing using Time 2 and Time 3 internal dimensions of university engagement, respectively. ^**^p < 0.01; ^***^p < 0.001.

For students' academic achievement at Time 4, the predicting effects of Time 1 personal well-being were partially mediated by only academic challenge at Time 2 and Time 3, with the estimated point of effect ranging between 0.09 and 0.10, but not learning with peers at either Time 2 or Time 3. Figures 3A,B depict the mediating effects of internal dimensions of university engagement regarding students' academic achievement.

Based on these results, students' individual well-being longitudinally predicted their learning achievement both directly and indirectly through the mediating effect of one or two internal dimensions of university engagement. Thus, Hypotheses 1 and 2 were largely supported.

**Figure 4:**
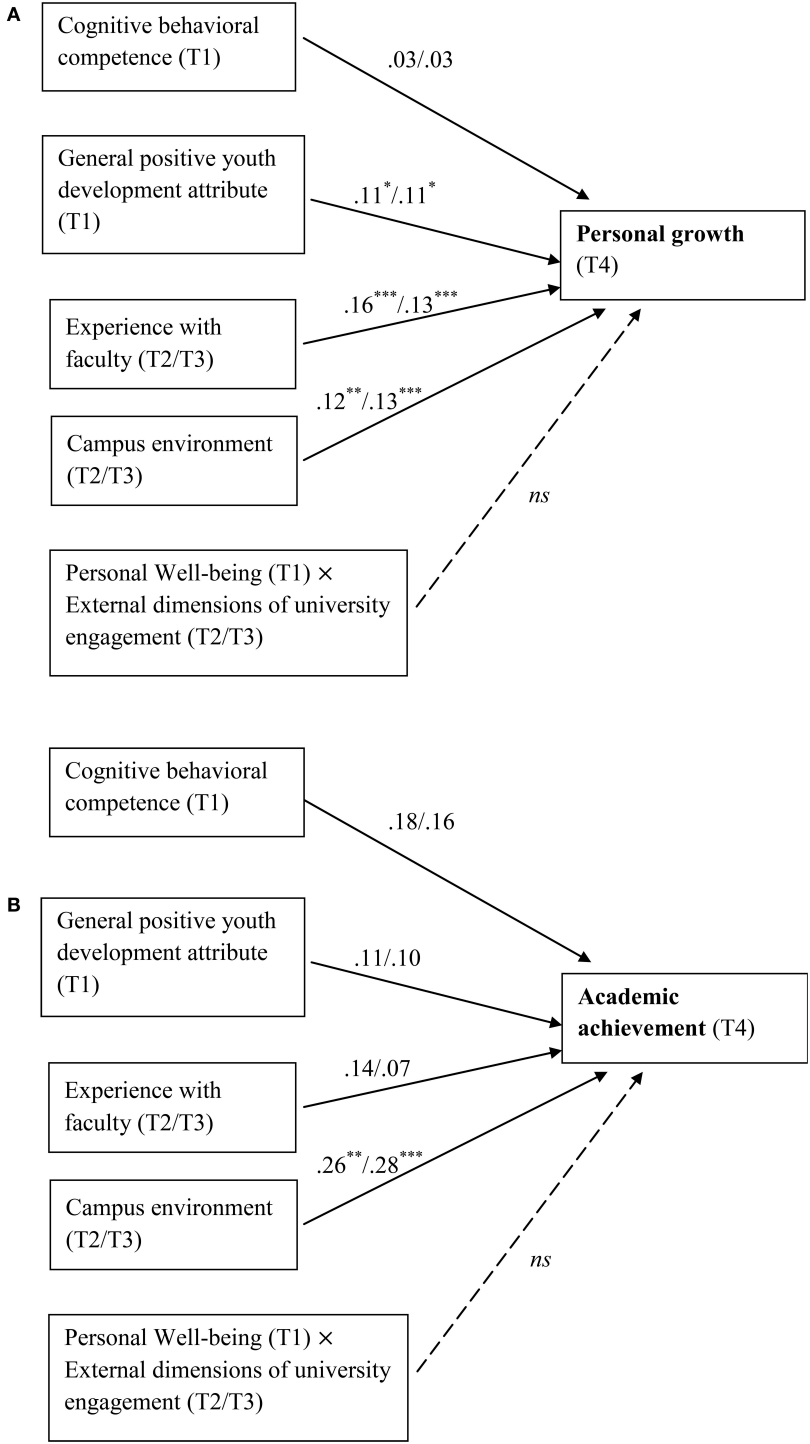
**(A)** Relationships among personal well-being, personal growth, and external dimensions of university engagement. T1 = Time 1; T2 = Time 2; T3 = Time 3; T4 = Time 4. The regression coefficients shown in the figure are unstandardized values with values before and after slash representing using Time 2 and Time 3 external dimensions of university engagement, respectively. **(B)** Relationships among personal well-being, academic achievement, and external dimensions of university engagement. T1 = Time 1; T2 = Time 2; T3 = Time 3; T4 = Time 4. The regression coefficients shown in the figure are unstandardized values with values before and after slash representing using Time 2 and Time 3 external dimensions of university engagement, respectively. ^*^p < 0.05; ^**^p < 0.01; ^***^p < 0.001.

Table 4 showed the results of testing moderation effects. First, after controlling the effects of the control variables, general PYD (β = 0.15, *p* = 0.002) but not cognitive-behavioral competence (β = 0.10, *p* = 0.169) had unique significant longitudinal predicting effect on students' personal growth, while both general PYD (β = 0.15, *p* = 0.037) and cognitive-behavioral competence (β = 0.16, *p* = 0.026) still significantly predicted academic achievement. Second, the two external dimensions of university engagement, experience with faculty and campus environment, at Time 2 and Time 3 both significantly predicted personal growth at Time 4 (β = 0.17−0.23, *p* < 0.01), but only campus environment at Time 2 (β = 0.18, *p* < 0.01) and Time 3 (β = 0.22, *p* < 0.001) significantly predicted academic achievement at Time 4. Hypothesis 3 was overall supported. However, the interactions between personal well-being and the two external dimensions of university engagement did not show significant effects on the two learning achievement measures. Hence, external dimensions of university engagement did not demonstrate significant moderating effects. Hypothesis 4 was not supported by the present results. Figures 4A,B illustrate the effect of external dimensions of university engagement on learning achievement.

**Table 4:** Regression analyses on personal growth and academic achievement at Time 4 (*N* = 425–427).

**Predictor**		**Personal growth (T4)**				**Academic achievement (T4)**			
		***B***	***SE***	**β**	**ΔR^2^**	***B***	***SE***	**β**	**ΔR^2^**
Step 1					0.09^***^				0.08^***^
	T1 Age	0.03	0.05	0.03		−0.10	0.10	−0.05	
	Gender^a^	−0.02	0.03	−0.04		−0.003	0.05	0.00	
	T1 Family intactness^b^	0.04	0.04	0.05		0.06	0.08	0.04	
	T1 Family economic status^c^	0.03	0.04	0.03		−0.07	0.08	−0.04	
	T1 CBC	0.06	0.04	0.10		0.19	0.09	0.16^*^	
	T1 GPYD	0.15	0.05	0.22^**^		0.22	0.10	0.15^*^	
Step 2					0.11^***^				0.07^***^
	T1 Age	0.04	0.04	0.04		−0.10	0.09	−0.05	
	Gender^a^	−0.03	0.02	−0.05		0.00	0.05	0.003	
	T1 Family intactness^b^	0.02	0.04	0.02		0.03	0.08	0.02	
	T1 Family economic status^c^	0.04	0.04	0.05		−0.03	0.08	−0.02	
	T1 CBC	0.03	0.04	0.05		0.18	0.09	0.14	
	T1 GPYD	0.11	0.05	0.15^*^		0.11	0.10	0.08	
	T2 EWF	0.16	0.03	0.23^***^		0.14	0.07	0.10	
	T2 CE	0.12	0.04	0.17^**^		0.26	0.08	0.18^**^	
	T1 CBC × T2 EWF	0.07	0.18	0.03		0.75	0.38	0.16	
	T1 GPYD × T2 EWF	0.01	0.17	0.01		−0.69	0.36	−0.15	
	T1 CBC × T2 CE	−0.23	0.12	−0.14		0.00	0.25	0.00	
	T1 GPYD × T2 CE	0.16	0.12	0.09		0.10	0.25	0.03	
Step 2					0.11^***^				0.07^***^
	T1 Age	−0.01	0.04	−0.01		−0.16	0.09	−0.08	
	Gender^a^	0.00	0.03	0.00		0.03	0.05	0.03	
	T1 Family intactness^b^	0.04	0.04	0.04		0.06	0.08	0.04	
	T1 Family economic status^c^	0.04	0.04	0.05		−0.01	0.08	−0.01	
	T1 CBC	0.03	0.04	0.05		0.16	0.09	0.12	
	T1 GPYD	0.11	0.05	0.15^*^		0.10	0.10	0.07	
	T3 EWF	0.13	0.04	0.20^***^		0.07	0.08	0.06	
	T3 CE	0.13	0.04	0.20^***^		0.28	0.08	0.22^***^	
	T1 CBC × T3 EWF	0.11	0.16	0.06		0.38	0.33	0.11	
	T1 GPYD × T3 EWF	−0.13	0.15	−0.07		−0.20	0.32	−0.06	
	T1 CBC × T3 CE	−0.12	0.13	−0.08		0.27	0.27	0.09	
	T1 GPYD × T3 CE	0.11	0.12	0.07		−0.44	0.26	−0.14	

a *: −1 = Female, 1 = male*.

b *: −1 = not intact, 1 = intact*.

c *: −1 = having economic disadvantage, 1 = without economic disadvantage. T1 = Time 1; T2 = Time 2; T3 = Time 3; T4 = Time 4. CBC, Cognitive behavioral competence; GPYD, General positive youth development attribute; EWF, Experience with faculty; CE, Campus environment*;

* *p < 0.05*;

** *p < 0.01*;

*** *p < 0.001*.

## Discussion

The present study tested and proved the hypothesis that students' personal well-being at the beginning of their university life significantly predicts their learning achievement in university. Results showed that students with a higher level of well-being in terms of positive youth development (PYD) competencies generally reported greater personal growth and achieved better accumulative GPA after 3 years of university study. These results echo previous findings showing that individual characteristics related to positive development are intimately associated with better learning achievement and less psychological problems (Caprara et al., 2011). For example, Durlak et al.'s (2011) meta-analysis concluded that psychosocial competence promotes academic performance in children and adolescents. Shek and Wu's (2016) recent paper showed the positive relationship as well. In this sense, the present longitudinal study lends further support to the impact of an individual's psychosocial competencies on one's subsequent development in university study. This finding suggests that students with higher levels of competencies tend to benefit more from university education and enjoy a more productive university life.

The approach we adopted in the present study to measure personal well-being was developed based on a PYD framework that included 15 constructs (e.g., social competence, behavioral competence, and resilience; Shek et al., 2007b). These constructs represent essential and common components used in effective PYD programs that promote positive youth outcomes and prevent youth problematic behaviors (Catalano et al., 2002). To further investigate whether development of these PYD attributes could contribute to students' outcomes in later years, researchers have conducted empirical longitudinal studies and gained supportive findings among middle school students (Sun and Shek, 2013; Yu and Shek, 2017). The present study extends such research into higher education context and suggests that enhancing positive developmental assets could be a promising strategy to promote student success in higher education.

In the present study, two composite indicators of personal well-being were considered: cognitive-behavioral competence and general PYD attributes. Apart from the common positive influence of these two constructs on students' learning achievement, the present study also showed a difference regarding the impacts. Specifically, after controlling the effects of other variables, the general PYD showed unique predicting effects on both self-report growth and academic achievement, whereas the cognitive-behavioral competence only demonstrated a unique predicting effect on academic achievement. Such a difference was a novel finding, because previous studies tended to calculate a global score for all positive development assets instead of using separate indicators (e.g., Sun and Shek, 2013). Our results did not suggest that cognitive-behavioral competence was not important in facilitating student learning. Instead, the results indicated that different positive attributes might have differential influences on student personal growth. Besides, as personal growth referred to not only cognitive aspects but also a broad area of social and interpersonal components (e.g., communication and civic responsibility), the general PYD that encompassed a variety of attributes might be a more effective predictor than the cognitive-behavioral competence which mainly involved cognitive attributes. Future studies need to examine these speculations by comparing the predicting effects of different positive development attributes on different aspects of student learning achievement.

The present findings regarding the longitudinal relationship between PYD competences and learning achievement have important educational application in Hong Kong. First, the findings suggest that the traditional belief held by many Chinese parents that adolescents will develop well as long as they can be admitted to a prestigious university is simply wrong. We found that students with lower level of personal well-being at the beginning of their university life showed lower learning achievements after 3 years of university study. Instead of merely focusing on adolescent academic development when preparing them for university life, more attention must be paid to adolescents' personal well-being. Second, regarding the answer to how students can actually benefit from higher education, considerable promise exists for enhancing student gains by promoting adolescent multiple psychosocial competences as suggested by the present study. While previous studies have proved that the development of multiple psychosocial competences can enhance students' academic performance in Western countries (Sklad et al., 2012; Gutman and Schoon, 2015), the present findings further support this conclusion by illustrating evidence in Chinese population. In fact, in view of the increasing trend of psychological problems among youth, scholars have suggested the establishment of multiple competences in young people through implementing curriculum-based PYD programs (Shek, 2010; Shek and Wong, 2011). Findings of the present study further consolidate the theoretical foundation for such a proposal.

Another research question of the present study was whether university engagement could mediate or moderate the longitudinal relationship between personal well-being and student learning achievement. As expected, the internal dimensions, especially academic challenge was a solid mediator. There are two significant implications of the findings. First, student internal engagement was a significant longitudinal predictor of student growth and academic achievement. In view of longitudinal impacts, the findings support an argument that being engaged in university establishes the foundation of dispositions and skills that are critical for students to live a prosperous and satisfactory life in the university and after graduation (Kuh, 2009b). In other words, students who are more engaged in university learning also perform better in developing habits of their mind and heart that in turn enhance their capability for continuous learning and development (Kuh, 2009b).

Despite that all aspects of university engagement were important for learning and development (Upadyaya and Salmela-Aro, 2013), academic challenge had strong predicting effects on both personal growth and academic achievement while learning with peers only predicted personal growth to some extent. Given that academic challenge mainly referred to student involvement in deep and independent learning and their use of different learning strategies, the effect of this engagement dimension on student learning achievement is self-evident: the more often students apply higher-order and multiple learning strategies in university learning, the more they tend to learn and the better they understand. Likewise, learning with peers, which mainly reflected students' cooperation or discussion with diverse others, may offer students opportunities to improve themselves by learning others' experiences. This is just like the positive influence of learning communities (Pike et al., 2011). Nevertheless, interaction with others may be more effective in promoting students' improvement in one area (e.g., social skills) than in another (e.g., cognitive or academic development).

The second finding related to the mediation effect was that students' personal competencies were significant longitudinal antecedents of their internal engagement during university years. This finding is consistent with previous results suggesting that students' confidence in their competence and self-efficacy were significant predictors of their university engagement (Fazey and Fazey, 2001; Llorens et al., 2007). Given that the transition year from secondary school to university is full of challenges and difficulties, the present finding implies that students who have developed important internal assets before college are more likely to cope with the transitional distress successfully, and to engage in a wide range of educationally productive activities. This would ultimately lead to a more productive university life (Trowler and Trowler, 2010; Li and Lerner, 2011). Once again, this finding highlights the importance of equipping youngsters with essential PYD competences, which has not yet received sufficient attention in Hong Kong.

While the mediating effect of internal dimensions of university engagement was significant, the moderating effect of external dimensions was not significant in the present study. There are several potential explanations. First, previous studies have found that some concepts (e.g., friend support) related to external dimensions of university engagement could significantly buffer the negative impacts of school stress while some others (e.g., global measure of social support) could not (Wilks and Spivey, 2010; Pidgeon et al., 2014). It is possible that the two external dimensions of university engagement measured in the present study (experience with faculty and campus environment), may not precisely capture the specific element that can effectively buffer the negative influence of lack of internal assets. For example, “friendship with teachers” which is of particular importance for Chinese students (Zhu et al., 2010), may not be adequately assessed by the current measure of university engagement which measures the overall interaction between students and faculty as well as other people on campus. Future studies need to further differentiate specific components of external dimensions of university engagement, such as peer support, teacher relationship, and institutional resources, and examine their respective roles in moderating the effects of personal attributes and learning achievement.

The second plausible explanation is that the long time span of the present study (i.e., 3 years) has weakened the moderating effect. Most previous studies that reported moderating effects of school environment were cross-sectional in nature. As such, future research may examine the moderating effects of external university engagement using both cross-sectional and longitudinal approaches. Nevertheless, it was found that the external dimensions of university engagement also had direct effect on students learning achievement. This means that institutions can facilitate student learning by creating supportive contextual environment such as providing resources or utilizing effective teaching pedagogy such as experiential and reflective teaching and learning methods, which can effectively deepen students' learning and critical thinking (Kolb and Kolb, 2005; Shek and Yu, 2017).

Although the effects of control variables are beyond the scope of the present study, several interesting observations are worth noting. First, no gender difference was observed regarding their internal engagement. This is inconsistent with previous finding (Yin and Wang, 2016) and suggests that female and male Chinese students of the present sample were equally engaged in university learning. Meanwhile, it was found that male students reported a lower level of external engagement than did female students only in the third year of university life (Time 3). This finding based on one time point cannot enable us to draw a conclusion on the gender effect in university engagement, yet it reminds us to consider gender factor when providing institutional and faculty support for students. For instance, pay more attention to male students' need in senior years. Second, regarding family intactness, although students coming from intact family tended to be more engaged in university learning at the second year, such an effect did not appear at the third year. This suggests that while unfavorable family environment exerts negative influence on youth development among secondary school students (Hopson and Lee, 2011; Yu and Shek, 2014), this influence may drop as students getting independence gradually and building diverse social networks in university. Besides, the support from and relationship with family members may play a more important role in university students' learning and performance than one's parental marital status (Cheng et al., 2012; Chang and Yang, 2016). Finally, students with family economic disadvantage perceived more supportive campus environment in the second and third year of university than those without economic disadvantage. This may be because students coming from poor family make more use of campus resources or they receive some specific help or support from the university. As the present study is a preliminary exploration in Hong Kong, the above findings need further validation and replication.

Despite the significant theoretical and practical implications, the present study has several limitations. The first limitation is on the measurement of learning achievement. The present study included a self-report measure (i.e., personal growth) and an objective measure (i.e., GPA), which produced similar results. Some researchers regarded self-report learning outcome measures as reliable as objective measures (Kuh, 2009b). In the present study, the scale used to measure personal growth was widely used in previous studies (Kuh, 2009b), and was developed to meet some conditions to ensure its validity and reliability. Besides, the scale showed good reliability (i.e., Cronbach's α was 0.90) in the current study. However, some researchers doubted that students may not always be able to accurately judge their own growth (Bowman and Green, 2013). In this sense, longitudinal assessments of student learning via objective tests would convey direct measures for personal growth, despite demanding requirements of financial and human resources. Nevertheless, to provide a full picture of student growth and improvement, future studies could apply both self-report measures and longitudinal comparisons between students' scores in objective achievement tests.

Second, the present study only involved students in one university in Hong Kong. To enhance the generalizability of findings, further research could involve students from different universities in Hong Kong and abroad (e.g., mainland China, other Asian countries). Third, the present study is quantitative research in nature, which is not able to provide in-depth information on how students differently cope with challenges and difficulties, as well as how they engage in university learning and utilize campus resources. Qualitative data could help researchers interpret and reflect on the findings. To gain a more comprehensive understanding about student development and its relationships to personal well-being and university engagement, multiple research methods including qualitative approaches should be used in future research. Nevertheless, this study is the first longitudinal study addressing several unanswered questions in the field. The findings can help educators and policy-makers to re-think about how the quality of academic life of university students can possibly be promoted.

## Ethics statement

The study has been approved by the Human Subjects Ethics Sub-committee (HSESC) (or its Delegate) of The Hong Kong Polytechnic University (Reference No.: HSEARS20130204003).

## Author contributions

DS and LY: designed the project and contributed to all steps of the work; LY and XZ: contributed to the data interpretation of the work and XZ drafted the work and revised it based on the critical comments provided by LY and DS; All authors approve of the final version of the manuscript and agree to be accountable for all aspects of the work in ensuring that questions related to the accuracy or integrity of any part of the work are appropriately investigated and resolved.

### Conflict of interest statement

The authors declare that the research was conducted in the absence of any commercial or financial relationships that could be construed as a potential conflict of interest.
